# The Efficacy and Surgical Outcomes of Cervical Foley Catheter Tourniquet in Placenta Accreta Spectrum Disorders

**DOI:** 10.3390/biomedicines14092095

**Published:** 2026-09-17

**Authors:** Huseyin Durukan, Hamza Yildiz, Kasim Akay, Ezgi Oktay, Faik Gurkan Yazici

**Affiliations:** 1 Department of Obstetrics and Gynecology, Faculty of Medicine, Mersin University, 33110 Mersin, Turkey; huseyindurukan@gmail.com (H.D.); gyazici70@yahoo.com (F.G.Y.); 2 Department of Obstetrics and Gynecology, Tarsus State Hospital, 33460 Mersin, Turkey; 3 Department of Obstetrics and Gynecology, Düziçi State Hospital, 80600 Osmaniye, Turkey; kasimakay57@gmail.com; 4 Department of Obstetrics and Gynecology, Medical Park TEM Hospital, 34260 Istanbul, Turkey; ezgioktaymd@gmail.com

**Keywords:** placenta accreta spectrum, foley catheter tourniquet, obstetric hemorrhage, cesarean delivery, hemostasis, uterine-sparing surgery

## Abstract

**Objective:** We aimed to evaluate the impact of the cervical Foley catheter tourniquet on perioperative surgical interventions, transfusion requirements, and maternal–neonatal outcomes in patients with placenta accreta spectrum (PAS). **Materials and Methods:** In this single-center retrospective cohort study, 265 patients with confirmed PAS were categorized into a tourniquet group (*n* = 87, cervical Foley catheter tourniquet applied after fetal delivery) and a control group (*n* = 178, standard hemostasis) following an institutional protocol update. Perioperative surgical interventions, hematologic parameters, transfusion needs, and maternal–neonatal outcomes were compared. **Results:** Baseline characteristics were generally comparable between groups. The decrease in hemoglobin (ΔHb) was significantly smaller in the tourniquet group than in controls (1.51 ± 1.11 vs. 1.75 ± 1.57 g/dL; *p* = 0.045). The tourniquet group had significantly fewer maternal ICU admissions (4.6% vs. 12.4%; *p* = 0.049); however, ICU length of stay among admitted patients did not differ significantly between groups (median 1.0 vs. 1.0 days; *p* = 0.585). The peripartum hysterectomy rate was lower in the tourniquet group (8.0% vs. 16.3%), although this difference did not reach conventional statistical significance (*p* = 0.085). Operative time was longer in the tourniquet group (105.0 vs. 60.0 min; *p* < 0.001). No maternal deaths occurred in either cohort. **Conclusions:** The cervical Foley catheter tourniquet is a feasible and low-cost surgical adjunct that was associated with a smaller perioperative decline in hemoglobin and lower maternal ICU admission rates, alongside higher rates of uterine-preserving procedures. However, because actual blood loss was not directly quantified and transfusion frequency remained high, its true independent effect on intraoperative blood loss and total transfusion requirements remains uncertain.

## 1. Introduction

Placenta accreta spectrum (PAS) encompasses a continuum of abnormally adherent and invasive placentation, ranging from placenta accreta to increta and percreta, and represents one of the most significant contributors to catastrophic obstetric hemorrhage and maternal morbidity worldwide [1]. The condition arises from a defect of the endometrial-myometrial interface, most commonly at the site of a prior cesarean scar, which permits abnormal trophoblastic invasion into or through the myometrium [1]. As cesarean delivery rates have risen globally, the incidence of PAS has risen in parallel, and it is now estimated to affect approximately 1 in 1000 deliveries in some populations [2].

Management of PAS is inherently high-risk: pooled data indicate that more than half of affected pregnancies culminate in peripartum hysterectomy and nearly half require blood transfusion for severe hemorrhage, with a nontrivial risk of maternal death [1,2]. To standardize diagnosis and communication of disease severity, the International Federation of Gynecology and Obstetrics (FIGO) introduced a clinical grading system ranging from Grade 1 (adherent placenta) to Grade 3 (percreta with variable degrees of extrauterine invasion) [3]. Optimal outcomes depend on antenatal diagnosis, delivery at a specialized multidisciplinary referral center, and a planned rather than emergent surgical approach, an approach that has been shown to reduce blood loss and transfusion requirements by as much as 50% in some series [1].

Despite this multidisciplinary framework, intraoperative hemorrhage control remains the central surgical challenge in PAS. Numerous adjunctive hemostatic techniques have been described, including internal iliac artery ligation or balloon occlusion, uterine artery embolization, B-Lynch and other compression sutures, and Bakri balloon tamponade; however, evidence supporting these adjuncts remains inconsistent, and systematic reviews and meta-analyses of prophylactic internal iliac artery balloon occlusion specifically have failed to demonstrate a consistent reduction in blood loss, transfusion requirement, or hysterectomy rate [4,5]. In this context, the use of a Foley catheter as a mechanical tourniquet around the lower uterine segment or cervix, first described by Ikeda et al. in 2005, has emerged as a simple, low-cost, and reproducible technique for achieving temporary occlusion of the uterine and collateral circulation before placental separation [6]. Subsequent case series and comparative studies have reported that this maneuver can reduce intraoperative blood loss, transfusion requirements, and certain surgical complications, while remaining technically feasible even outside high-resource settings [7,8].

Nevertheless, most of the available literature on the Foley catheter tourniquet technique consists of small case series or single-arm cohorts, and comparative data evaluating its impact on a broad range of maternal and neonatal outcomes within a large, single-center PAS population remain limited. Mersin University Faculty of Medicine Hospital serves as the regional referral center for the antenatal diagnosis and surgical management of PAS in southern Turkey, and it adopted the cervical Foley catheter tourniquet technique as a standard component of its surgical protocol for PAS from 2022 onward. This temporal shift in institutional practice provided an opportunity to retrospectively compare perioperative and postoperative outcomes between patients managed with and without this technique. The aim of the present study was therefore to evaluate the clinical associations and perioperative surgical outcomes of the cervical Foley catheter tourniquet regarding hemostatic interventions, transfusion requirements, and maternal–neonatal morbidity in a large single-center cohort of patients with PAS.

## 2. Materials and Method

### 2.1. Study Design and Setting

This retrospective cohort study was conducted at Mersin University Faculty of Medicine Hospital, a tertiary care center that serves as the regional referral center for the antenatal diagnosis and surgical management of placenta accreta spectrum (PAS) disorders. The study was approved by the Institutional Ethics Committee of Mersin University Faculty of Medicine (approval no. 2026/447, dated 5 August 2026) and was performed in accordance with the principles of the Declaration of Helsinki. Informed consent was obtained from all individual participants included in the study. The medical records of all patients who underwent cesarean delivery with an antenatal and/or intraoperative diagnosis of PAS between 2013 and 2026 were retrospectively reviewed.

Because the cervical Foley catheter tourniquet was adopted as an institutional routine beginning in January 2022, group allocation was determined chronologically rather than by randomization. Consequently, this investigation essentially compares two consecutive eras of institutional PAS management (the pre-tourniquet era 2013–2021 versus the tourniquet era 2022–2026), with calendar time representing an inherent potential confounder.

### 2.2. Study Population and Grouping

A total of 265 patients with intraoperatively and/or histopathologically confirmed PAS (placenta accreta, increta, or percreta) were included in the analysis. Patients were categorized into two groups according to whether a Foley catheter tourniquet was used to control hemorrhage during surgery. As the Foley catheter tourniquet technique was adopted as a standard institutional protocol from January 2022 onward, patients who delivered before this date constituted the control group (*n* = 178, no tourniquet applied), whereas patients who delivered on or after this date constituted the tourniquet group (*n* = 87). Group allocation therefore reflected a temporal change in institutional surgical practice rather than randomization. Patients with incomplete medical records, multiple gestation, or postpartum hemorrhage attributable to a cause other than PAS were excluded.

To ensure diagnostic homogeneity and avoid misclassification, PAS diagnosis was established based on a standardized three-tiered diagnostic pathway comprising antenatal ultrasonography, intraoperative clinical assessment, and histopathological examination:

1. Antenatal Ultrasound Assessment: All suspected patients underwent standardized transabdominal and transvaginal grayscale and color Doppler ultrasonography performed by experienced maternal-fetal medicine specialists. Antenatal sonographic criteria followed standardized European Working Group on Abnormally Invasive Placenta (EW-AIP) and FIGO consensus terminology, including loss of the retroplacental clear zone, presence of irregular intraplacental lacunae with turbulent flow, bladder wall interruption, and uterovesical hypervascularity.

2. Intraoperative Diagnostic Criteria: An intraoperative clinical diagnosis of PAS was strictly defined according to predefined FIGO clinical criteria rather than subjective difficulty in placental delivery. Specifically, failure of spontaneous placental separation, difficult manual removal alone, or non-specific bleeding without anatomical invasion were not considered diagnostic of PAS. Intraoperative confirmation strictly required direct macroscopic evidence of abnormal invasion into the uterine wall, characterized by (a) persistent adherence of placental tissue to the basal plate with absence of a cleavage plane despite gentle traction; (b) localized or diffuse hypervascularity, engorged vessels, and thinning/purplish discoloration of the lower uterine segment; or (c) direct placental invasion through the myometrium breaching the uterine serosa or involving adjacent pelvic structures (bladder/parametrium) for placenta percreta.

3. Histopathological Confirmation: Formal histopathological confirmation was performed in all cases where resected tissue or whole organs were available (namely all peripartum hysterectomies and segmental myometrial resections). Specimens were reviewed by institutional perinatal pathologists according to standardized guidelines, assessing the microscopic absence of intervening decidua and direct invasion of chorionic villi into the myometrium (accreta), within the myometrium (increta), or extending to/through the serosa (percreta).

### 2.3. Cervical Foley Catheter Tourniquet Technique

In the tourniquet group, following delivery of the fetus via a standard or high hysterotomy and subsequent exteriorization of the uterus, a 16-8 F Foley catheter is securely tied around the infundibulopelvic ligament and lower uterine segment, encompassing both ovaries and fallopian tubes (without damaging the fallopian tubes and fimbriae), temporarily occluding uterine and collateral arterial blood flow to the lower segment and placental bed. This maneuver provided a clear, relatively bloodless surgical field for subsequent placental separation, focal myometrial resection, and placement of intrauterine hemostatic compression sutures. Following definitive surgical hemostasis, the tourniquet was unclamped, adequate reperfusion of the lower segment was confirmed, and the catheter was completely removed. In the control group, cesarean delivery was performed without this maneuver, and hemostasis was achieved using conventional techniques (compression sutures, uterine devascularization, balloon tamponade, or peripartum hysterectomy), as clinically indicated (Video S1).

### 2.4. Data Collection and Variables

Demographic and obstetric data (maternal age, gravida, parity, number of prior miscarriages and curettages, number of previous cesarean sections and vaginal deliveries, mode of conception, gestational age at delivery, birth weight, and indication for cesarean delivery), preoperative hematologic parameters, intraoperative hemostatic interventions (intrauterine hemostatic suture, uterine lower segment resection, Bakri balloon tamponade, internal iliac artery ligation, B-Lynch compression suture, pelvic/uterine packing, and peripartum hysterectomy), operative time, transfusion requirements (packed red blood cells, fresh frozen plasma, total blood products, and massive transfusion defined as ≥4 units), postoperative hemoglobin and hematocrit values and their perioperative change (ΔHb, ΔHct), maternal intensive care unit (ICU) admission and length of stay, postoperative hospital length of stay, neonatal Apgar scores at 1 and 5 min, and postoperative complications (reoperation, urinary tract injury, maternal infection, and maternal mortality) were retrospectively extracted from the hospital electronic medical records. Institutional transfusion protocols remained standardized across the entire study duration. The intraoperative decision to transfuse packed red blood cells (PRBCs) was clinically guided by active ongoing surgical hemorrhage, hemodynamic instability (mean arterial pressure < 65 mmHg or refractory tachycardia), or an anticipated drop in hemoglobin below 7–8 g/dL in accordance with national obstetric hemorrhage guidelines. Postoperative complete blood count indices (hemoglobin and hematocrit) were routinely obtained approximately 6 h following the completion of all intraoperative and immediate postoperative blood product transfusions to allow for hemodynamic and fluid equilibration. In non-transfused patients, blood samples were drawn within 6 to 12 h postoperatively. Transfusion thresholds, laboratory turnaround protocols, and institutional indications for blood component therapy did not undergo structural protocol changes after 2022.

Total operative time was defined as the duration in minutes from initial skin incision to complete skin closure as recorded in anesthesiology operative logs. The specific procedural time dedicated exclusively to the placement, adjustment, and removal of the cervical Foley catheter tourniquet was not recorded as an isolated variable in the retrospective electronic charts.

### 2.5. Statistical Analysis

Statistical analyses were performed using IBM SPSS Statistics for Windows, version 23.0 (IBM Corp., Armonk, NY, USA). The normality of distribution of continuous variables was assessed, and as none followed a normal distribution, they are presented as median (25th–75th percentile) and mean ± standard deviation, having been compared between groups using the Mann–Whitney U test. Categorical variables are presented as number (percentage) and were compared using the Pearson chi-square test, or Fisher’s exact test when the expected cell count was less than 5. A two-tailed *p*-value < 0.05 was considered statistically significant.

To address potential temporal confounding and baseline imbalances between the historical cohorts, multivariable logistic regression analyses were conducted for primary categorical outcomes (maternal ICU admission, peripartum hysterectomy, and blood transfusion requirement). Covariates included in the models were maternal age, gestational age at delivery, preoperative hemoglobin, number of prior cesarean sections, number of prior miscarriages, and emergency cesarean indication. Adjusted odds ratios (aORs) with 95% confidence intervals (CIs) were calculated. Multivariable linear regression was performed to evaluate independent predictors of perioperative hemoglobin decrease (ΔHb).

## 3. Results

### 3.1. Demographic and Baseline Clinical Characteristics

**Figure 1:**
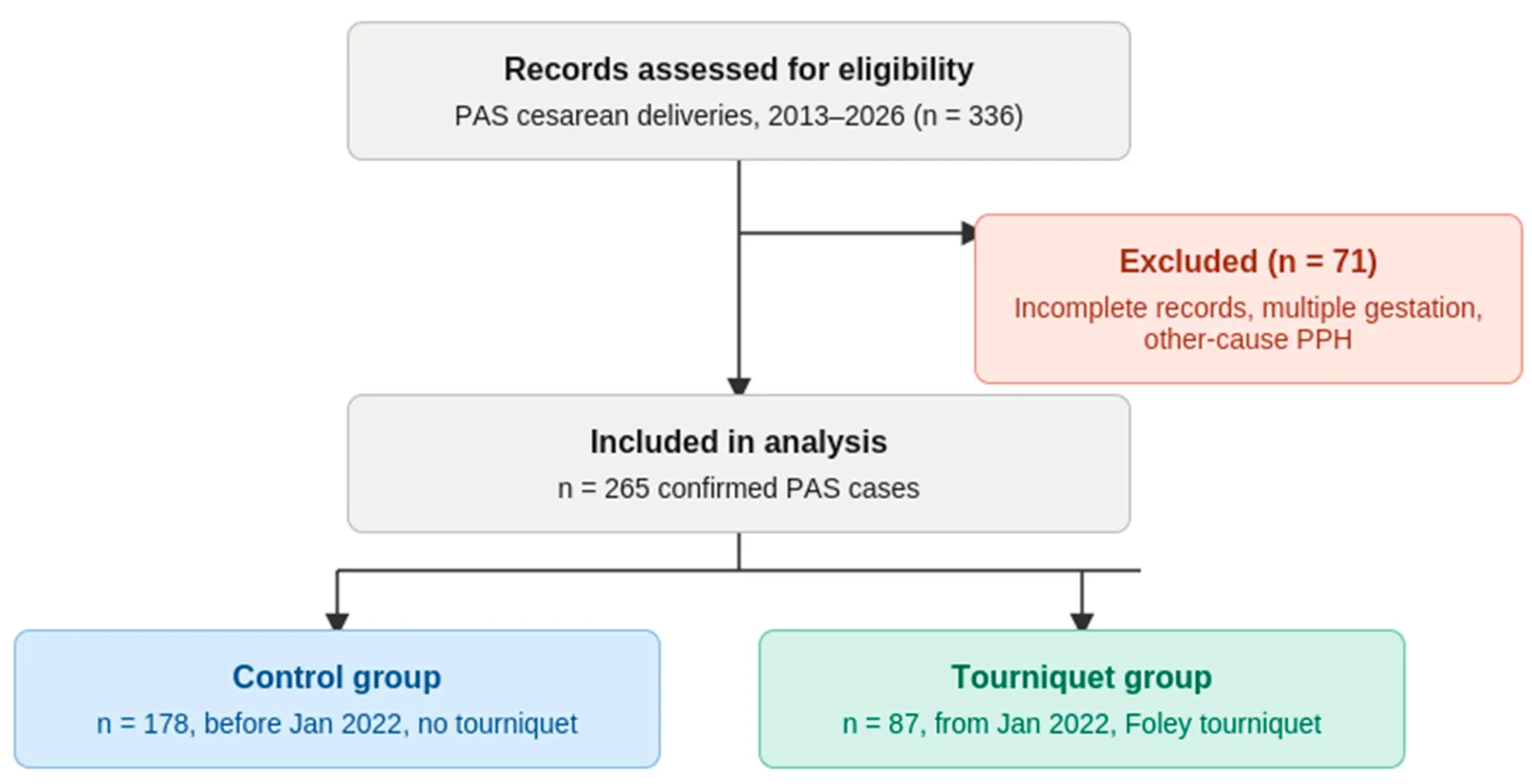
Study flow diagram.

A total of 265 patients with PAS were analyzed: 87 (32.8%) in the tourniquet group (≥2022) and 178 (67.2%) and in the control group (<2022, no tourniquet)) (Table 1, Figure 1). Maternal age, gravida, parity, number of curettages, number of previous cesarean sections, number of previous vaginal deliveries, and birth weight were comparable between the groups (*p* > 0.05 for all). The number of prior miscarriages was higher in the tourniquet group than in the control group (0.72 ± 1.22 vs. 0.37 ± 0.74; *p* = 0.024). Gestational age at delivery was significantly earlier in the tourniquet group (37.0 (35.6–37.5) weeks) compared with the control group (37.3 (36.0–38.1) weeks; *p* = 0.014). Spontaneous conception was less frequent in the tourniquet group (90.8% vs. 98.3%; *p* = 0.004). Preoperative hemoglobin (10.88 ± 1.19 vs. 11.22 ± 1.38 g/dL; *p* = 0.035) and hematocrit (32.14 ± 3.33% vs. 33.62 ± 3.79%; *p* = 0.001) were significantly lower in the tourniquet group. The rate of emergency cesarean delivery did not differ significantly between groups (40.2% vs. 48.9%; *p* = 0.192).

Among the 36 patients who underwent peripartum hysterectomy with definitive pathological examination, the distribution of PAS severity did not differ significantly between the tourniquet and control groups (placenta accreta: 57.1% (*n* = 4) vs. 24.1% (*n* = 7); placenta increta: 14.3% (*n* = 1) vs. 34.5% (*n* = 10); placenta percreta: 14.3% (*n* = 1) vs. 13.8% (*n* = 4); other indications: 14.3% (*n* = 1) vs. 27.6% (*n* = 8); *p* = 0.367). For the overall cohort, surgical complexity and indirect markers of severe myometrial invasion were reflected by significantly higher rates of lower uterine segment resection (35.6% vs. 14.6%, *p* < 0.001) and intrauterine hemostatic suturing (69.0% vs. 39.9%, *p* < 0.001) in the tourniquet cohort.

Regarding markers of anatomical PAS severity and surgical complexity, all patients in both groups presented with complete placenta previa (100.0%). Anterior placental localization overlying the uterine scar was significantly more frequent in the tourniquet group than in historical controls (54.0% (*n* = 47) vs. 29.2% (*n* = 52), *p* < 0.001), reflecting a higher anatomical risk for severe lower segment neovascularization and dense myometrial adherence. The number of prior cesarean sections was numerically higher in the tourniquet group (mean 1.57 ± 1.24 vs. 1.29 ± 1.13, *p* = 0.073). Rates of antenatal PAS suspicion (89.7% vs. 88.2%, *p* = 0.725) and suspected bladder invasion requiring surgical repair (3.4% vs. 6.7%, *p* = 0.398) were comparable between groups. Histopathological examination of hysterectomy specimens (Table 2) showed no significant difference in the distribution of accreta, increta, and percreta (*p* = 0.367).

### 3.2. Perioperative Surgical Interventions and Hemostatic Procedures

The frequency of intrauterine hemostatic suture (69.0% vs. 39.9%; *p* < 0.001) and uterine lower segment resection (35.6% vs. 14.6%; *p* < 0.001) was significantly higher in the tourniquet group than in the control group (Table 2). No significant between-group differences were found in the rates of Bakri balloon tamponade (43.7% vs. 38.2%; *p* = 0.424), internal iliac artery ligation (51.7% vs. 46.6%; *p* = 0.513), B-Lynch compression suture (6.9% vs. 3.9%; *p* = 0.365), pelvic/uterine packing (0.0% vs. 1.1%; *p* = 1.000), or peripartum hysterectomy (8.0% vs. 16.3%; *p* = 0.085). Operative time was significantly longer in the tourniquet group (105.0 (75.0–120.0) minutes) than in the control group (60.0 (60.0–120.0) minutes; *p* < 0.001).

The peripartum hysterectomy rate was numerically lower in the tourniquet group than in the control group (8.0% (7/87) vs. 16.3% (29/178)), reflecting an interesting numerical reduction that did not achieve conventional statistical significance in univariate analysis (*p* = 0.085). Given the total of 36 hysterectomy events across the cohort, this comparison was likely underpowered to detect smaller effect sizes.

### 3.3. Transfusion Requirements, Laboratory Parameters, and Maternal/Neonatal Outcomes

The perioperative decrease in hemoglobin (ΔHb) was significantly smaller in the tourniquet group compared with controls (1.51 ± 1.11 vs. 1.75 ± 1.57 g/dL; *p* = 0.045), whereas the decrease in hematocrit (ΔHct) did not reach statistical significance (*p* = 0.098) (Table 3). However, a significantly higher proportion of patients in the tourniquet cohort received a blood transfusion (78.2% vs. 61.2%; *p* = 0.008), while total units transfused, packed red blood cells, fresh frozen plasma, and the rate of massive transfusion (≥4 units: 33.3% vs. 31.5%; *p* = 0.780) did not significantly differ between cohorts. Maternal ICU admission was significantly less frequent in the tourniquet group than in the control group (4.6% (*n* = 4) vs. 12.4% (*n* = 22); *p* = 0.049). When analyzing the duration of intensive care strictly among admitted patients (*n* = 26), the ICU length of stay was comparable between the tourniquet group (median 1.0 (IQR 1.0–1.25) days, mean 1.25 ± 0.50) and the control group (median 1.0 (IQR 1.0–2.0) days, mean 1.68 ± 1.13; *p* = 0.585). Postoperative hospital length of stay was significantly longer in the tourniquet group (2.89 ± 1.16 vs. 2.71 ± 1.41 days; *p* = 0.026). Neonatal Apgar scores at both 1 min (6.04 ± 1.76 vs. 6.97 ± 1.71; *p* < 0.001) and 5 min (7.94 ± 1.31 vs. 8.54 ± 1.34; *p* < 0.001) were significantly lower in the tourniquet group. No significant differences were observed between groups in the rates of reoperation (0.0% vs. 1.7%; *p* = 0.553), urinary tract injury (3.4% vs. 6.7%; *p* = 0.398), or maternal infection (0.0% vs. 2.2%; *p* = 0.306). No maternal deaths occurred in either group.

In unadjusted comparisons, neonatal Apgar scores at 1 min (6.04 ± 1.76 vs. 6.97 ± 1.71; *p* < 0.001) and 5 min (7.94 ± 1.31 vs. 8.54 ± 1.34; *p* < 0.001) were significantly lower in the tourniquet group. In multivariable linear regression adjusting for gestational age at delivery, emergency cesarean indication, and maternal age, gestational age remained a strong independent determinant of both 1-min (Beta = 0.229, *p* < 0.001) and 5-min Apgar scores (Beta = 0.214, *p* < 0.001), while emergency cesarean delivery significantly lowered 1-min Apgar scores (Beta = −0.529, *p* = 0.023).

### 3.4. Multivariable Adjusted Analysis

In multivariable logistic regression adjusting for maternal age, gestational age, preoperative hemoglobin, prior cesareans, prior miscarriages, and emergency delivery (Table 4), the use of a cervical Foley catheter tourniquet was associated with significantly lower adjusted odds of maternal ICU admission (aOR = 0.196, 95% CI: 0.056–0.686, *p* = 0.011). In addition, tourniquet application was associated with reduced adjusted odds of peripartum hysterectomy (aOR = 0.247, 95% CI: 0.088–0.696, *p* = 0.008). However, in view of the limited number of hysterectomy events (*n* = 36) and the non-significant univariate comparison (*p* = 0.085), this finding should be interpreted cautiously as a hypothesis-generating trend rather than definitive proof of risk reduction.

In contrast, the higher crude transfusion rate observed in the tourniquet group did not remain statistically significant in the multivariable model (aOR = 1.866, 95% CI: 0.935–3.724, *p* = 0.077), where lower baseline hemoglobin (aOR = 0.540, 95% CI: 0.418–0.697, *p* < 0.001) and prior cesarean deliveries (aOR = 2.263, 95% CI: 1.634–3.135, *p* < 0.001) were the primary independent drivers of transfusion need. In multivariable linear regression evaluating independent predictors of perioperative hemoglobin decrease (ΔHb), tourniquet utilization was not an independent predictor of ΔHb (unstandardized B = −0.182, 95% CI: −0.564 to 0.200, *p* = 0.349), with baseline hemoglobin and units transfused serving as the major determinants.

In additional logistic regression models adjusting for baseline covariates, tourniquet utilization was independently associated with a significantly higher likelihood of performing lower uterine segment resection (aOR = 2.385, 95% CI: 1.205–4.723, *p* = 0.013) and intrauterine hemostatic suturing (aOR = 3.478, 95% CI: 1.954–6.191, *p* < 0.001).

## 4. Discussion

In this retrospective single-center cohort of 265 patients with PAS, the use of a cervical Foley catheter tourniquet was associated with several distinct perioperative and postoperative outcome differences compared with the historical control group managed without this maneuver. Patients in the tourniquet group more frequently underwent intrauterine hemostatic suturing and uterine lower segment resection, had a smaller perioperative decline in hemoglobin, and experienced fewer and shorter ICU admissions, yet they also had a longer operative time, a higher proportion requiring blood transfusion, and a longer postoperative hospital stay.

The core physiological rationale for the cervical Foley catheter tourniquet, first described by Ikeda et al., is that circumferential compression of the lower uterine segment and cervix mechanically occludes the uterine and collateral arterial supply, thereby providing a relatively bloodless field for placental separation and definitive hemostasis [6]. Several subsequent case series have supported the feasibility and hemostatic benefit of this maneuver, including its use in emergency, prenatally undiagnosed, or resource-limited settings [8,9,10]. The smaller ΔHb decrease and lower ICU admission rate observed in our tourniquet cohort were consistent with previous observational reports suggesting favorable perioperative hemodynamic stability, although the lack of directly quantified blood loss precludes definitive claims of superior hemostatic efficacy [11].

Although a smaller decrease in hemoglobin (ΔHb) was observed in the tourniquet group, interpreting this metric as definitive evidence of superior hemostatic efficacy requires substantial caution. A higher proportion of patients in the tourniquet cohort received blood transfusions (78.2% vs. 61.2%, *p* = 0.008). Because postoperative hemoglobin levels were assessed approximately 6 h following the completion of blood transfusions, the smaller ΔHb observed in this group is, at least in part, an artifact of transfusion-mediated volume and red cell replacement rather than a reflection of solely diminished intraoperative blood loss. In the absence of gravimetric or volumetric quantification of intraoperative blood loss, ΔHb cannot serve as an isolated marker of surgical hemostasis in heavily transfused cohorts.

The longer operative time (median 105 vs. 60 min, *p* < 0.001) and higher crude transfusion rate (78.2% vs. 61.2%, *p* = 0.008) in the tourniquet group must be interpreted in light of both procedural characteristics and anatomical complexity. Clinically, anterior placenta previa overlying a prior hysterotomy scar was documented in over half of the tourniquet cohort compared to 29.2% in controls (*p* < 0.001), creating an inherently more vascular and challenging operative field. Rather than proceeding directly to cesarean hysterectomy, surgeons in the tourniquet cohort undertook technically demanding uterine-sparing interventions—demonstrated by significantly higher rates of lower uterine segment resection (35.6% vs. 14.6%) and intrauterine hemostatic suturing (69.0% vs. 39.9%). Performing meticulous lower segment excision and multilayer hemostatic reconstruction naturally requires substantially more operative time than standard closure or rapid hysterectomy. Furthermore, after multivariable adjustment for baseline anemia and prior scar burden (Table 4), tourniquet placement was not an independent driver of transfusion (aOR = 1.866, *p* = 0.077), clarifying that the greater crude transfusion frequency reflected lower baseline hemoglobin rather than surgical failure of the tourniquet.

However, our finding of a longer operative time and a higher proportion of patients requiring transfusion in the tourniquet group contrasts with the shorter procedure duration reported by Abouda et al. [11]. This discrepancy most likely reflects differences in baseline disease severity rather than an inherent limitation of the technique itself. Patients in our tourniquet group had significantly lower preoperative hemoglobin and hematocrit, a higher number of prior miscarriages, and a lower rate of spontaneous conception, all of which may be surrogate markers of more extensive placental invasion or a more complex surgical field. In our primary analysis, we observed higher rates of lower uterine segment resection and hemostatic suturing alongside lower baseline hematologic values in the tourniquet group, which could clinically suggest management of more complex or deeper placental invasions. However, examination of histopathologically confirmed hysterectomy specimens did not demonstrate a statistically significant shift in the proportions of accreta, increta, and percreta between cohorts (*p* = 0.367). A major challenge in evaluating PAS severity in contemporary cohorts is that conservative, uterine-preserving approaches—which increased substantially in the tourniquet era—preclude full-thickness histopathological grading. Therefore, while surgical complexity was markedly elevated in the tourniquet group, as evidenced by reconstructive procedures, whether this reflects true differences in pathological invasion depth or evolving institutional enthusiasm for conservative uterine reconstruction remains an important limitation.

Regarding critical care utilization, tourniquet application was independently associated with a lower rate of maternal ICU admission. However, when evaluating length of stay exclusively among patients who required intensive care, the duration of ICU hospitalization did not significantly differ between cohorts (median 1.0 vs. 1.0 days, *p* = 0.585). Rather than demonstrating a direct physiological prevention of hemodynamic collapse, this observation is associative and may also reflect contemporaneous shifts in institutional ICU admission thresholds, anesthesia management, or evolving critical care pathways between the two eras [4]. Taken together, these observations reinforce the notion that no single adjunctive maneuver, including the cervical Foley catheter tourniquet, can be expected to eliminate the hemorrhagic risk inherent to PAS surgery, and that outcomes are likely most favorably influenced by the combination of antenatal diagnosis, individualized surgical planning, and multidisciplinary team care emphasized in current consensus guidelines [1,12].

Furthermore, multivariable models for maternal ICU admission (*n* = 26 total events) and peripartum hysterectomy (*n* = 36 total events) are subject to potential model overfitting and limited precision, as reflected by relatively wide confidence intervals. Therefore, these adjusted risk estimates should be interpreted with appropriate caution.

The trend toward a lower peripartum hysterectomy rate in the tourniquet group, although not statistically significant, may be hypothesis-generating. By providing a controlled, relatively bloodless field, the tourniquet may facilitate more conservative, uterus-preserving interventions, such as focal myometrial resection with hemostatic suturing, in patients who might otherwise have proceeded directly to hysterectomy. This possibility is consistent with data from the PACCRETA study, which demonstrated that conservative management of PAS can be associated with lower maternal morbidity than cesarean hysterectomy when appropriately selected [13]. Larger, adequately powered studies are needed to determine whether the tourniquet technique independently increases the feasibility of uterine conservation in PAS. Consequently, demonstrated an independent reduction in peripartum hysterectomy (aOR = 0.247), but highlight an encouraging trend requiring verification in larger multicenter trials.

The significantly lower 1-min and 5-min Apgar scores observed in the tourniquet group (6.04 vs. 6.97 and 7.94 vs. 8.54, respectively; *p* < 0.001) warrant careful clinical consideration. Because the cervical Foley catheter tourniquet is placed strictly following the delivery of the neonate and umbilical cord clamping, this mechanical intervention has no direct physiological impact on the fetus. Rather, this finding is multifactorial. First, gestational age at delivery was significantly earlier in the tourniquet group compared with historical controls (median 37.0 vs. 37.3 weeks, *p* = 0.014). Second, and more importantly, recent evidence has demonstrated that in pregnancies complicated by placenta previa, the presence of confirmed placenta accreta spectrum is independently associated with adverse neonatal respiratory outcomes and increased respiratory support requirements, even after rigorously adjusting for gestational age at delivery [14]. The lower baseline hemoglobin and higher procedural complexity observed in our tourniquet cohort suggest a population with greater clinical and placental severity, which, combined with earlier iatrogenic delivery, likely accounts for the lower immediate neonatal vigor and Apgar scores rather than any aspect of the intraoperative tourniquet maneuver itself [1,15]. Notably, real-world compliance with this recommended delivery window remains suboptimal even in expert centers, and delivery timing itself is an independent determinant of neonatal outcome in PAS pregnancies [15]. Importantly, 1-min and 5-min Apgar scores are influenced by multiple maternal, fetal, and intrapartum factors—including gestational maturity, maternal sedation, and operative delivery speed—and should not be interpreted as an equivalent or direct surrogate of fetal acidemia or metabolic distress. As previously demonstrated, low Apgar scores show limited sensitivity and specificity for objective cord blood acidosis (pH < 7.00 or base excess), and umbilical cord blood gas analysis remains the reference standard for assessing intrapartum hypoxia. Because umbilical arterial cord gas analysis was not systematically obtained across historical cases, these lower Apgar scores primarily reflect earlier delivery and procedural complexity rather than verified neonatal hypoxia.

Several limitations should be considered. This was a retrospective, single-center, non-randomized comparison in which group allocation was determined by a historical change in surgical protocol rather than by patient-level randomization, introducing the possibility of temporal confounding from concurrent advances in anesthetic, transfusion, and perioperative care. Blood loss was not directly quantified but inferred from hemoglobin and hematocrit changes and transfusion data. Second, intraoperative blood loss was not directly or volumetrically quantified, but rather inferred from perioperative hemoglobin changes and transfusion records. Given that postoperative hemoglobin was measured 6 h post-transfusion, the observed ΔHb was inherently influenced by administered blood products, confounding the ability to isolate the true mechanical hemostatic contribution of the tourniquet. Baseline differences in preoperative hematologic status and obstetric history between groups further limit direct causal inference regarding the tourniquet’s independent effect. Strengths of this study include its relatively large sample size for a single PAS referral center, a clearly described and reproducible surgical technique, and comprehensive capture of maternal and neonatal outcomes. Prospective, multicenter studies with standardized blood loss quantification and adjustment for disease severity are needed to more precisely define the independent contribution of the cervical Foley catheter tourniquet to maternal and neonatal outcomes in PAS.

We acknowledge that diagnostic classification across a long observational period may introduce potential ascertainment bias, particularly between patients undergoing hysterectomy (with full-thickness histological confirmation) and those managed conservatively. To minimize diagnostic heterogeneity, we strictly excluded patients with simple retained placenta or bleeding due to uterine atony and applied standardized FIGO intraoperative criteria across both study periods. Although tissue-level pathological confirmation is inherently unavailable in patients undergoing uterus-preserving surgery without myometrial excision, combining objective macroscopic criteria with high antenatal ultrasound concordance provides strong clinical fidelity across both historical and contemporary cohorts.

Given the historical nature of the control cohort spanning from 2013 to 2021 compared with the 2022–2026 tourniquet cohort, temporal confounding from evolving surgical experience and perioperative protocols is an important consideration. To overcome this limitation, multivariable adjusted regression analyses were performed. Crucially, Foley tourniquet placement retained a significant independent protective association against maternal ICU admission (aOR = 0.196) and demonstrated an independent reduction in peripartum hysterectomy (aOR = 0.247) when controlling for key clinical covariates including prior cesarean deliveries and baseline hemoglobin. Additionally, multivariable modeling demonstrated that the higher raw transfusion frequency in the tourniquet cohort was primarily driven by lower baseline hemoglobin and surgical history rather than tourniquet application itself.

Importantly, intraoperative blood loss was not directly or gravimetrically quantified; rather, hemorrhagic outcomes were inferred from perioperative hemoglobin changes and transfusion records. Because postoperative hematologic indices are heavily modified by intravenous crystalloid resuscitation, intraoperative hemodilution, and timing of blood transfusions relative to phlebotomy, ΔHb cannot serve as an equivalent surrogate for directly measured blood loss.

The primary methodological limitation of this study is the historical-control design and the substantial temporal confounding inherent to comparing two distinct eras of care. The tourniquet cohort (2022–2026) was managed in an era characterized by mature institutional multidisciplinary protocols, enhanced antenatal ultrasound diagnostic precision, specialized surgical teams, refined massive transfusion protocols, and advanced critical care pathways compared with the earlier control cohort (2013–2021). Furthermore, institutional attitudes toward uterine-preserving procedures significantly evolved over the 14-year timeframe, which likely contributed to the higher frequencies of lower uterine segment resection and hemostatic suturing observed in the later period independently of the tourniquet device itself. Although multivariable logistic regression was utilized to adjust for baseline imbalances and temporal trends, residual unmeasured confounding from chronological changes in institutional philosophy, surgical expertise, and anesthetic management cannot be completely eliminated. Therefore, the observed favorable surgical and critical care outcomes should be interpreted as reflecting the evolution of comprehensive PAS care in the modern era with the tourniquet as an adjunctive component, rather than establishing isolated device efficacy.

Third, standardized FIGO clinical grading was not systematically documented in electronic medical records throughout the earlier historical period (2013–2016), precluding a universal retrospective FIGO severity score across the entire 14-year cohort. Moreover, full-thickness histopathological confirmation could only be obtained in patients undergoing hysterectomy or partial myometrial resection, as conservative preservation inherently leaves the placental bed intact.

Regarding maternal safety, we observed no maternal deaths in either cohort, and rates of reoperation (0.0% vs. 1.7%), urinary tract injury (3.4% vs. 6.7%), and postoperative infection (0.0% vs. 2.2%) did not significantly differ between groups (Table 3). However, the absence of a statistically significant difference cannot be interpreted as definitive proof of procedural safety. Given the low absolute event rates of major maternal visceral injuries and infections in contemporary practice, our sample size is inherently underpowered to detect uncommon but clinically critical adverse events. Therefore, our findings should be interpreted cautiously as demonstrating that no significant increase in the measured postoperative complications was observed in this cohort.

## 5. Conclusions

In this single-center retrospective cohort of patients with placenta accreta spectrum, the application of a cervical Foley catheter tourniquet was associated with a smaller perioperative hemoglobin decline and a lower frequency of maternal ICU admission, alongside higher utilization of uterine-preserving procedures. The observed reduction in peripartum hysterectomy represents an encouraging, hypothesis-generating trend. No increase in major maternal complications—including urinary tract injury, infection, or reoperation—was observed. These findings support the cervical Foley catheter tourniquet as a feasible, low-cost surgical adjunct that is associatively linked with favorable perioperative outcomes in modern PAS management. Given the historical-control design, residual confounding cannot be excluded, and large prospective multicenter trials with objective blood loss quantification are warranted to confirm these clinical associations.

## Figures and Tables

**Table 1:** Comparison of Demographic, Clinical, and Baseline Obstetric Characteristics Between Groups.

Variable	Tourniquet Group (*n* = 87)	Control Group (*n* = 178)	*p*-Value
Maternal Age (years) ^a^	31.0 (28.0–36.0) [32.02 ± 6.29]	32.0 (28.0–36.0) [32.27 ± 5.30]	0.546
Gravida ^a^	3.0 (2.0–5.0) [3.60 ± 2.12]	3.0 (2.0–4.0) [3.28 ± 1.65]	0.425
Parity ^a^	2.0 (1.0–3.0) [1.84 ± 1.38]	2.0 (1.0–2.0) [1.72 ± 1.25]	0.500
Number of Miscarriages ^a^	0.0 (0.0–1.0) [0.72 ± 1.22]	0.0 (0.0–1.0) [0.37 ± 0.74]	0.024 *
Number of Curettages ^a^	0.0 (0.0–0.0) [0.07 ± 0.37]	0.0 (0.0–0.0) [0.14 ± 0.43]	0.101
Number of Previous Cesarean Sections ^a^	1.0 (1.0–2.0) [1.57 ± 1.24]	1.0 (0.0–2.0) [1.29 ± 1.13]	0.073
Number of Previous Vaginal Deliveries ^a^	0.0 (0.0–0.0) [0.28 ± 0.74]	0.0 (0.0–0.0) [0.46 ± 0.93]	0.053
Gestational Age at Delivery (weeks) ^a^	37.0 (35.6–37.5) [36.17 ± 2.38]	37.3 (36.0–38.1) [36.67 ± 2.05]	0.014 *
Birth Weight (g) ^a^	3000 (2670–3350) [2963 ± 628]	3010 (2590–3300) [2923 ± 563]	0.625
Emergency Cesarean Indication ^b^	35 (40.2%)	87 (48.9%)	0.192
Spontaneous Pregnancy ^b^	79 (90.8%)	175 (98.3%)	0.004 *
Preoperative Hemoglobin (g/dL) ^a^	10.8 (10.2–11.6) [10.88 ± 1.19]	11.2 (10.3–12.1) [11.22 ± 1.38]	0.035 *
Preoperative Hematocrit (%) ^a^	32.0 (30.0–35.0) [32.14 ± 3.33]	33.6 (31.0–36.0) [33.62 ± 3.79]	0.001 *
**Baseline Clinical/Severity Parameter**	**Tourniquet Group** **(*n* = 87)**	**Control Group** **(*n* = 178)**	***p*** **-value**
Placenta Previa Type (Total Previa) ^a^	87 (100.0%)	178 (100.0%)	1.000
Placental Location ^a^			< 0.001 *
Anterior placenta (scar overlying)	47 (54.0%)	52 (29.2%)	
Posterior placenta	32 (36.8%)	39 (21.9%)	
Lateral placenta	4 (4.6%)	11 (6.2%)	
Indeterminate/not documented	4 (4.6%)	76 (42.7%)	
Previous Cesarean Deliveries ^b^	Median (IQR): 1.0 (1.0–2.0) Mean ± SD: 1.57 ± 1.24	Median (IQR): 1.0 (0.0–2.0) Mean ± SD: 1.29 ± 1.13	0.073
Suspected Bladder Involvement/Injury	3 (3.4%)	12 (6.7%)	0.398
Antenatal PAS Suspicion ^a^	78 (89.7%)	157 (88.2%)	0.725

Continuous data are presented as median (25th–75th percentile) [mean ± standard deviation]; categorical data are presented as *n* (%). ^a.^ Continuous variables were compared using the Mann–Whitney U test data presented as median (IQR) [mean ± SD]; ^b.^ categorical variables were compared using the Pearson chi-square test. * *p* < 0.05, statistically significant.

**Table 2:** Comparison of Perioperative Surgical Interventions and Hemostatic Procedures.

Surgical/Pathological Variable	Tourniquet Group (*n* = 87)	Control Group (*n* = 178)	*p*-Value
**Surgical & Hemostatic Interventions**			
Intrauterine Hemostatic Suture ^a^	60 (69.0%)	71 (39.9%)	<0.001 *
Uterine Lower Segment Resection ^a^	31 (35.6%)	26 (14.6%)	<0.001 *
Bakri Balloon Tamponade ^a^	38 (43.7%)	68 (38.2%)	0.424
Hypogastric Artery Ligation (IIAL) ^a^	45 (51.7%)	83 (46.6%)	0.513
B-Lynch Compression Suture ^a^	6 (6.9%)	7 (3.9%)	0.365
Pelvic/Uterine Packing ^b^	0 (0.0%)	2 (1.1%)	1.000
Peripartum Hysterectomy ^b^	7 (8.0%)	29 (16.3%)	0.085
Operative Time (minutes) ^c^	Median (IQR): 105.0 (75.0–120.0) Mean ± SD: 102.36 ± 27.84	Median (IQR): 60.0 (60.0–120.0) Mean ± SD: 83.66 ± 32.86	<0.001 *
**Histopathological Subtypes (in Hysterectomy Specimens, *n* = 36) ^b^** Overall *p* = 0.367			
Placenta Accreta	4 (57.1%)	7 (24.1%)	0.367
Placenta Increta	1 (14.3%)	10 (34.5%)	0.367
Placenta Percreta	1 (14.3%)	4 (13.8%)	0.367
Other/Unclassified Indication ^d^	1 (14.3%)	8 (27.6%)	0.367

Categorical data are presented as *n* (%); operative time is presented as median (IQR) [mean ± SD]. ^a^ Categorical variables were compared using the Pearson chi-square test. ^b^ Categorical variables were compared using Fisher’s exact test when expected cell counts were <5. ^c^ Continuous variable was compared using the Mann–Whitney U test. ^d^ Specimens from peripartum hysterectomies performed primarily for catastrophic bleeding due to severe total previa, uterine rupture, or intractable uterine atony without definitive transmural myometrial invasion. * *p* < 0.05, statistically significant.

**Table 3:** Transfusion Requirements, Laboratory Parameters, and Maternal/Postoperative Outcomes.

Clinical/Laboratory Parameter	Tourniquet Group (*n* = 87)	Control Group (*n* = 178)	*p*-Value
ΔHb (Preop Hb − Postop Hb) (g/dL) ^a^	1.5 (0.6–2.5) [1.51 ± 1.11]	1.9 (1.0–2.8) [1.75 ± 1.57]	0.045 *
ΔHct (Preop Hct − Postop Hct) (%) ^a^	4.0 (2.0–8.0) [4.90 ± 3.49]	6.0 (3.0–8.0) [5.40 ± 4.50]	0.098
Postoperative Hemoglobin (g/dL) ^a^	9.3 (8.7–9.9) [9.37 ± 1.03]	9.3 (8.6–10.2) [9.47 ± 1.25]	0.636
Postoperative Hematocrit (%) ^a^	27.0 (25.0–29.0) [27.24 ± 3.10]	28.0 (25.8–30.1) [28.22 ± 3.78]	0.043 *
Total Blood Transfusion (PRBCs + Whole Blood) (Units) ^a^	2.0 (1.0–4.0) [3.03 ± 2.82]	2.0 (0.0–5.0) [2.81 ± 4.24]	0.096
Packed Red Blood Cells (PRBCs) (Units) ^a^	2.0 (0.0–3.0) [1.98 ± 1.89]	1.0 (0.0–4.0) [1.95 ± 2.14]	0.479
Fresh Frozen Plasma (FFP) (Units) ^a^	1.0 (0.0–2.0) [1.02 ± 1.05]	0.0 (0.0–2.0) [1.20 ± 1.63]	0.679
Patients Receiving Blood Transfusion ^b^	68 (78.2%)	109 (61.2%)	0.008 *
Massive Transfusion Requirement (≥4 Units) ^b^	29 (33.3%)	56 (31.5%)	0.780
Maternal ICU Admission ^c^	4 (4.6%)	22 (12.4%)	0.049 *
Maternal ICU Admission ^b,c^	4 (4.6%)	22 (12.4%)	0.049 *
ICU Length of Stay among Admitted Patients (days) ^a^	**(*n* = 4)** Median (IQR): 1.0 (1.0–1.25) Mean ± SD: 1.25 ± 0.50	**(*n* = 22)** Median (IQR): 1.0 (1.0–2.0) Mean ± SD: 1.68 ± 1.13	0.585
Postoperative Hospital Length of Stay (days) ^a^	3.0 (2.0–3.0) [2.89 ± 1.16]	2.0 (2.0–3.0) [2.71 ± 1.41]	0.026 *
Apgar Score, 1 min ^a^	6.0 (5.0–7.0) [6.04 ± 1.76]	7.0 (6.0–8.0) [6.97 ± 1.71]	<0.001 *
Apgar Score, 5 min ^a^	8.0 (7.0–9.0) [7.94 ± 1.31]	9.0 (8.0–9.0) [8.54 ± 1.34]	<0.001 *
Reoperation Requirement ^c^	0 (0.0%)	3 (1.7%)	0.553
Urinary Tract Injury ^c^	3 (3.4%)	12 (6.7%)	0.398
Maternal Infection ^c^	0 (0.0%)	4 (2.2%)	0.306
Maternal Mortality	0 (0.0%)	0 (0.0%)	–

ΔHb and ΔHct represent the difference between preoperative and postoperative values. Continuous variables were compared using the Mann–Whitney U test (^a^); categorical variables were compared using the Pearson chi-square test (^b^), or Fisher’s exact test (^c^) when expected cell counts were <5. * *p* < 0.05.

**Table 4:** Multivariable Logistic Regression Analysis for Primary Clinical Outcomes.

Dependent Outcome	Variable	Adjusted OR (aOR)	95% Confidence Interval	*p*-Value
Maternal ICU Admission	Foley Tourniquet Use	0.196	0.056–0.686	0.011 *
	Maternal Age (per year)	1.112	1.014–1.218	0.023 *
	Gestational Age (per week)	1.003	0.804–1.252	0.978
	Preoperative Hb (per g/dL)	0.846	0.612–1.169	0.311
	Prior Cesarean Delivery	1.791	1.260–2.545	0.001 *
	Prior Miscarriages	1.449	0.954–2.199	0.082
	Emergency Cesarean	1.642	0.602–4.482	0.333
Peripartum Hysterectomy	Foley Tourniquet Use	0.247	0.088–0.696	0.008 *
	Maternal Age (per year)	1.075	0.993–1.164	0.074
	Gestational Age (per week)	1.191	0.937–1.515	0.154
	Preoperative Hb (per g/dL)	0.721	0.533–0.974	0.033 *
	Prior Cesarean Delivery	2.098	1.499–2.936	<0.001 *
	Prior Miscarriages	1.305	0.889–1.915	0.174
	Emergency Cesarean	1.177	0.477–2.908	0.724
Blood Transfusion Requirement	Foley Tourniquet Use	1.866	0.935–3.724	0.077
	Preoperative Hb (per g/dL)	0.540	0.418–0.697	<0.001 *
	Prior Cesarean Delivery	2.263	1.634–3.135	<0.001 *

* *p* < 0.05, statistically significant. aOR: Adjusted Odds Ratio.

## Data Availability

The datasets used and/or analyzed during the current study are available from the corresponding author on reasonable request.

## Supplementary Materials

The following supporting information can be downloaded at https://www.mdpi.com/article/10.3390/biomedicines14092095/s1, Video S1: Video of PAS.

## References

[1] Ghosh A., Lee S., Lim C., Vogelzang R.L., Chrisman H.B. (2023). Placenta Accreta Spectrum: An Overview. Semin. Interv. Radiol..

[2] Carusi D.A. (2018). The placenta accreta spectrum: Epidemiology and risk factors. Clin. Obstet. Gynecol..

[3] Jauniaux E., Ayres-de-Campos D., Langhoff-Roos J., Fox K.A., Collins S., Diagnosis F.P.A., Panel M.E.C., Duncombe G., Klaritsch P., Chantraine F. (2019). FIGO classification for the clinical diagnosis of placenta accreta spectrum disorders. Int. J. Gynecol. Obstet..

[4] Liang D., Zhao H., Liu D., Lin Y. (2021). Internal iliac artery balloon occlusion in the management of placenta accreta: A systematic review and meta-analysis. Eur. J. Radiol..

[5] Nankali A., Salari N., Kazeminia M., Mohammadi M., Rasoulinya S., Hosseinian-Far M. (2021). The effect prophylactic internal iliac artery balloon occlusion in patients with placenta previa or placental accreta spectrum: A systematic review and meta-analysis. Reprod. Biol. Endocrinol..

[6] Ikeda T., Sameshima H., Kawaguchi H., Yamauchi N., Ikenoue T. (2005). Tourniquet technique prevents profuse blood loss in placenta accreta cesarean section. J. Obstet. Gynaecol. Res..

[7] Leanza C. Prevenzione e Trattamento dell ‘Emorragia Postpartum (EPP) con una Nuova Tecnica di Posizionamento del Balloon Intrauterino Tipo Bakr: Analisi dei Risultati Ottenuti Presso la SC di Ginecologia ed Ostetricia dell’Azienda Ospedaliera Universitaria di Alessandria. https://hdl.handle.net/20.500.14238/647.

[8] Adarkwa K.O., Adu-Bredu T., Djokoto K.R. (2024). Foley’s catheter tourniquet: A lifesaving, innovative, inexpensive, and useful tool in reducing blood loss in prenatally undiagnosed placenta accreta spectrum surgery. Eur. J. Obstet. Gynecol. Reprod. Biol..

[9] Staniczek J., Manasar-Dyrbuś M., Skowronek K., Winkowska E., Stojko R. (2023). Foley catheter as a tourniquet for peripartum hemorrhage prevention in patients with placenta accreta spectrum—A two case report and a review of the literature. Medicina.

[10] Staniczek J., Manasar-Dyrbuś M., Winkowska E., Skowronek K., Stojko R. (2023). Foley Catheter as a Tourniquet for Hemorrhage Prevention During Peripartum Hysterectomy in Patients with Placenta Accreta Spectrum (PAS)—A Hospital-Based Study. Life.

[11] Abouda H.S., Marzouk S.B., Boussarsar Y., Aloui H., Frikha H., Hammami R., Chennoufi B., Maghrebi H. (2024). Tourniquet on the low segment of the uterus reduces blood loss in postpartum hemorrhage during hysterectomy for placenta accreta: Old but gold. Eur. J. Obstet. Gynecol. Reprod. Biol. X.

[12] Jauniaux E., Hussein A.M., Fox K.A., Collins S.L. (2019). New evidence-based diagnostic and management strategies for placenta accreta spectrum disorders. Best Pract. Res. Clin. Obstet. Gynaecol..

[13] Sentilhes L., Seco A., Azria E., Beucher G., Bonnet M.-P., Branger B., Carbillon L., Chiesa C., Crenn-Hebert C., Dreyfus M. (2022). Conservative management or cesarean hysterectomy for placenta accreta spectrum: The PACCRETA prospective study. Am. J. Obstet. Gynecol..

[14] Mlodawska M., Zmelonek-Znamirowska A., Swiercz G., Mehta J., Mlodawski J. (2026). Neonatal Respiratory Outcomes in Placenta Previa with Histopathologically Confirmed Placenta Accreta Spectrum. J. Clin. Med..

[15] Salmanian B., Einerson B.D., Carusi D.A., Shainker S.A., Nieto-Calvache A.J., Shrivastava V.K., Subramaniam A., Zuckerwise L.C., Lyell D.J., Khandelwal M. (2022). Timing of delivery for placenta accreta spectrum: The Pan-American Society for the Placenta Accreta Spectrum experience. Am. J. Obstet. Gynecol. MFM.

